# Fatty Acids Abolish *Shigella* Virulence by Inhibiting Its Master Regulator, VirF

**DOI:** 10.1128/spectrum.00778-23

**Published:** 2023-05-04

**Authors:** Rita Trirocco, Martina Pasqua, Angela Tramonti, Milena Grossi, Bianca Colonna, Alessandro Paiardini, Gianni Prosseda

**Affiliations:** a Institute Pasteur Italia, Department of Biology and Biotechnologies “Charles Darwin”, Sapienza University of Rome, Rome, Italy; b Institute of Molecular Biology and Pathology, National Research Council, Rome, Italy; c Department of Biochemical Sciences, Sapienza University of Rome, Rome, Italy; Centre national de la recherche scientifique, Aix-Marseille Université

**Keywords:** bacterial pathogens, *Shigella* virulence, AraC-XylS regulators, fatty acids

## Abstract

The pathogenicity of *Shigella*, the intracellular pathogen responsible for human bacillary dysentery, depends on a coordinated and tightly regulated expression of its virulence determinants. This is the result of a cascade organization of its positive regulators, with VirF, a transcriptional activator belonging to the AraC-XylS family, in a pivotal position. VirF itself is submitted to several well-known regulations at the transcriptional level. In this work, we present evidence for a novel posttranslational regulatory mechanism of VirF mediated by the inhibitory interaction with specific fatty acids. By homology modeling and molecular docking analyses, we identify a jelly roll motif in the structure of ViF capable of interacting with medium-chain saturated and long-chain unsaturated fatty acids. *In vitro* and *in vivo* assays show that capric, lauric, myristoleic, palmitoleic, and sapienic acids interact effectively with the VirF protein, abolishing its transcription-promoting activity. This silences the virulence system of *Shigella*, leading to a drastic reduction in its ability to invade epithelial cells and proliferate in their cytoplasm.

**IMPORTANCE** In the absence of a valid vaccine, the main therapeutic approach currently used to treat shigellosis is based on the use of antibiotics. The emergence of antibiotic resistance jeopardizes the future effectiveness of this approach. The importance of the present work resides both in the identification of a new level of posttranslational regulation of the *Shigella* virulence system and in the characterization of a mechanism offering new opportunities for the design of antivirulence compounds, which may change the treatment paradigm of *Shigella* infections by limiting the emergence of antibiotic-resistant bacteria.

## INTRODUCTION

*Shigella* is a Gram-negative facultative intracellular pathogen, closely related to Escherichia coli and divided into four subspecies (S. dysenteriae, S. flexneri, S. boydii, and S. sonnei). Counting up to about 165 million yearly cases worldwide, *Shigella* is regarded as a major bacterial cause of severe diarrhea. Although usually self-limiting, shigellosis can be fatal in children in Africa and South Asia (1). The infection occurs through person-to-person transmission via the fecal-oral route or via ingestion of contaminated food or water containing as low as 10 to 100 bacterial cells (2).

The *Shigella* virulence program is fully activated in the human colon. The intestinal mucosa is invaded, triggering debilitating diarrhea, through a complex multistep process. First, *Shigella* moves from the intestinal lumen by transcytosis through M cells and is released into an intraepithelial pocket. Here, macrophages engulf the bacterium, which secures its survival by inducing pyroptosis of the infected macrophages. This step is associated with the release of several proinflammatory cytokines, critical mediators of an acute and massive inflammatory response (3, 4). *Shigella* is now free to invade the epithelial cells from the basolateral pole, using a type III secretion system (T3SS), and spread to adjacent cells (5). This sophisticated strategy requires the coordinated expression of several genes located on the chromosome as well as on a large virulence plasmid (pINV) (6). Most genes required for host cell invasion and macrophage killing are contained in the so-called “entry region” of the pINV, a 31-kb conserved region arranged as a pathogenicity island (PAI)-like structure and consisting of 34 genes organized into two large, divergently transcribed clusters. This region encodes a specific T3SS, including its effector proteins (Ipa) and the corresponding chaperones (7), and also contains two regulatory genes, *virB*, and *mxiE*, which encode regulators responsible for the sequential expression of most *Shigella* virulence genes (8, 9). Other genes encoding proteins relevant to the invasive process, namely, *icsA*, *phoN2*, *ospG*, *ipaHs*, and *virF_30_* (here, simply VirF), are disseminated along the rest of the pINV (10–14). The expression of the virulence genes is organized as a three-level cascade. First, VirF directly activates the *icsA* and *virB* genes; then, VirB activates the transcription of the genes responsible for the assembly of T3SS, its effectors, and *mxiE*; and finally, the MxiE protein activates genes typically expressed during the intracellular step of *Shigella* infection (15, 16).

The VirF protein is the master controller of the entire virulence system, and, given its pivotal role, many studies have been focused on its regulation, leading to the characterization of multiple mechanisms that contribute to its stringent regulation (15, 17–21). VirF is a member of the AraC-XylS family of transcriptional activators, which are characterized by a 99- to 100-amino-acid segment containing two HTH motifs separated by an alpha helix (22–24). The HTH motifs are required for DNA binding and together constitute the DNA binding domain (DBD). In most AraC-XylS members, the DBD is at the C terminus, and an additional N-terminal domain, typically referred to as “companion domain” (CD), can be present and is usually involved in protein dimerization and ligand binding (25). Based on phylogenetic and functional criteria, VirF has been included in a subgroup of AraC-XylS proteins, together with Vibrio cholerae ToxT, enterotoxigenic E. coli (ETEC) Rns, and Salmonella enterica RtsA, HilC, and HilD (26). These proteins are known for their significant role in the virulence expression of the corresponding pathogens and are inhibited by specific ligands, most notably fatty acids (27–33). This phenomenon has been related to the geospatial programming of virulence expression mediated by the metabolites that these enteropathogens encounter during their journey through the gut (34).

Based on these observations, we asked whether also *Shigella* virulence could be affected by the interaction of VirF with compounds met in the intestinal environment. To test this hypothesis, we performed an accurate structural prediction of the VirF protein and then selected, by molecular docking analysis, a group of fatty acids as candidate ligands. We demonstrate that some of these molecules can effectively suppress *Shigella* virulence, both *in vivo* and *in vitro*, by interacting with the VirF protein, thus inhibiting its transcriptional-promoting activity on the *virB* gene, whose product is responsible for triggering the expression of the major effectors of the *Shigella* virulence (35) and thereby silencing the whole virulence system.

## RESULTS

### Structure modeling of VirF and docking studies with fatty acids.

**FIG 1:**
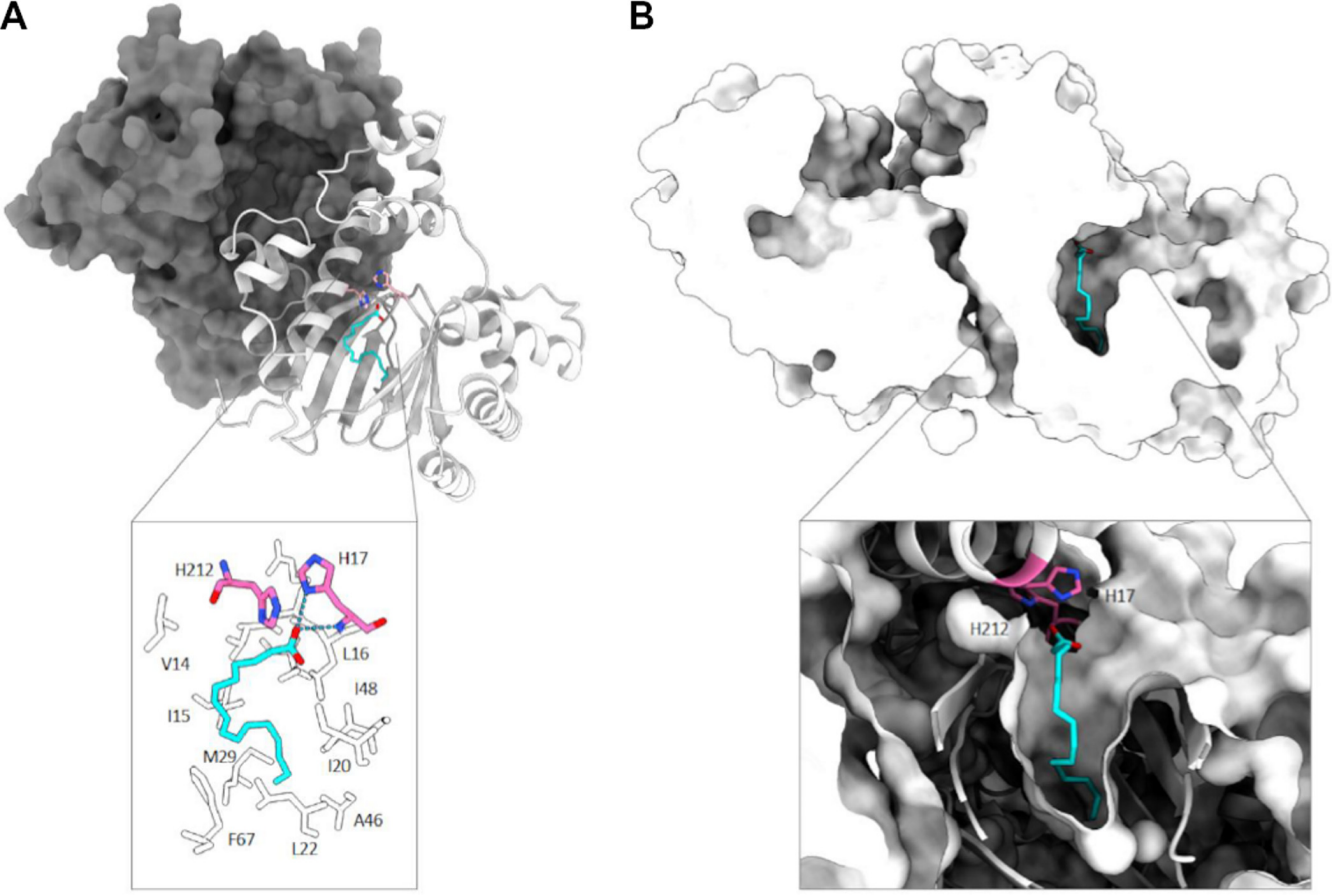
Structure of VirF in complex with a fatty acid. (A) The dimeric model of VirF is shown, with one monomer represented as a gray surface and the other one as a white cartoon. Palmitoleic acid is represented as cyan sticks. The residues interacting with the ligand are represented in the panel. (B) Representation of the cavities of VirF and the cavity accommodating the ligand are represented in the panel.

To obtain structural insights into the activation and modulation of VirF by small molecules, we modeled its structure using the homologous transcriptional regulator Rns as a template (PDB accession no. 6XIU [31]; percentage of identity, 41%). Initial modeling, refinement with AlphaFold2, and validation allowed us to obtain a final model whose metrics (Ramachandran outliers, energy plots, etc.) are close to a low-resolution crystal structure, i.e., 3.0 Å (see Fig. S1 in the supplemental material). The model shows that VirF maintains the two typical domains of the homologous AraC family proteins, i.e., a small ligand companion domain (CD, residues 1 to 159) and a DNA binding domain (DBD; residues 160 to 262). The latter is composed of seven alpha-helices with several evolutionarily conserved, positively charged residues exposed to the solvent and potentially involved in DNA binding (Arg174, Arg176, Arg192, Arg210, Arg242, Lys253, and Lys254). The N-terminal domain (residues 1 to 159) folds in a jelly roll motif made up of eight β-strands and four α-helices (Fig. 1A). The crystal structure of the Rns protein contains a moiety of decanoic acid within the hydrophobic cleft of the N-terminal domain. Given the structural similarity of Rns and VirF, we hypothesized that, similar to Rns, also VirF could bind fatty acids (FAs), and we initially modeled the decanoic acid within the cleft, using molecular docking methods (see Materials and Methods). Pockets of Rns and VirF model are mostly hydrophobic except for the polar side chains (identified in VirF as His17 and His212) (Fig. 1A, detail) involved in binding to the carboxyl group of the fatty acid. However, in VirF, the cleft appears larger than in Rns and is not completely filled by decanoic acid, suggesting that VirF can accommodate bulkier hydrophobic FAs. Indeed, the size of the cleft (~275 Å^3^) (Fig. 1B) is closer to that of another member of the AraC family, ToxT (PDB accession no. 3GBG), whose structure was solved in complex with its palmitoleate ligand (28). In addition, the residues of the ToxT, Rns, and VirF proteins that interact with the carboxylic part of the FAs (Lys31-Lys230 in ToxT, His20-Arg75 in Rns, and His17-His212 in VirF) are not conserved, nor is the expected orientation of the aliphatic part of the ligands in the hydrophobic pocket of VirF compared to the orientation in Rns or ToxT (not predicted for Rns and ToxT).

Altogether, these data suggest that different FAs may interact with ToxT, Rns, and VirF and/or that the same fatty acid may interact with different degrees of binding affinity (expressed as predicted dissociation constant). Therefore, by molecular docking analysis, we identified a pool of FAs potentially able to interact with VirF with different binding affinity and belonging to four chemical groups, medium-chain saturated fatty acids (MC-SFAs), long-chain saturated fatty acids (LC-SFAs), medium-chain unsaturated fatty acids (MC-UFAs), and long-chain unsaturated fatty acids (LC-UFAs). The MC-SFAs predicted interactors are capric (CA) and lauric (LA) acids, the LC-SFAs are myristic (MY), palmitic (PA), stearic (ST), and arachidic (AR) acids, the only representative of the MC-UFAs group is decenoic acid, and the LC-UFAs group comprises myristoleic (MYO), palmitoleic (PAO), sapienic (SA), and oleic (OL) acids (Table 1).

**TABLE 1:** Estimated energy of interaction (rerank score [62]) by docking of FAs in VirF

Common name	Structure	Carbon atoms:double bonds	Energy
Capric acid	CH_3_(CH_2_)_8_COOH	10:0	−63.9
Lauric acid	CH_3_(CH_2_)_10_COOH	12:0	−70.2
Myristic acid	CH_3_(CH_2_)_12_COOH	14:0	−81.8
Palmitic acid	CH_3_(CH_2_)_14_COOH	16:0	−97.0
Stearic acid	CH_3_(CH_2_)_16_COOH	18:0	−75.9
Arachidic acid	CH_3_(CH_2_)_18_COOH	20:0	−49.1
Decenoic acid	CH=CH(CH2)_7_COOH	10:1	−67.3
Myristoleic acid	CH_3_(CH_2_)_3_CH=CH(CH2)_7_COOH	14:1	−112.3
Palmitoleic acid	CH_3_(CH_2_)_5_CH=CH(CH2)_7_COOH	16:1	−116.8
Oleic acid	CH_3_(CH_2_)_7_CH=CH(CH2)_7_COOH	18:1	−100.2
Sapienic acid	CH_3_(CH_2_)_8_CH=CH(CH2)_4_COOH	16:1	−119.4

### S. flexneri virulence gene expression following treatment with fatty acids.

**FIG 2:**
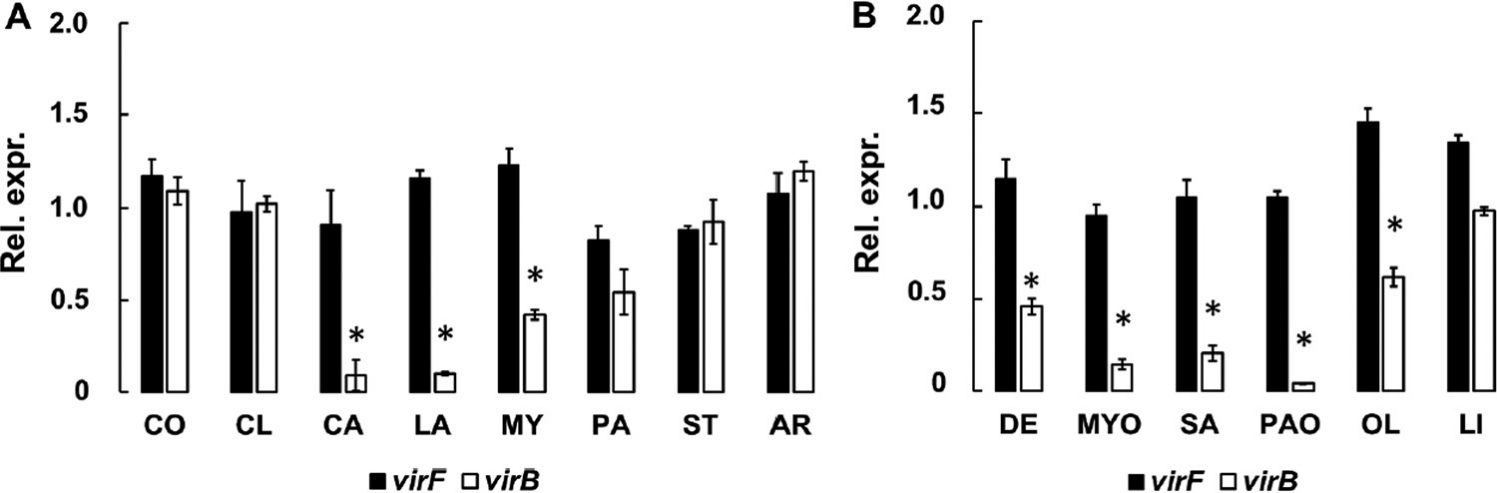
FAs reduce *virB* transcription without significatively affecting *virF* gene transcription. The graph shows the relative expression levels of *virF* and *virB* genes in S. flexneri strain M90T grown in the presence of 0.02% selected MC- and LC-SFAs (A) and MC- and LC-UFAs (B). Data were obtained by RT-qPCR, and values are compared to the corresponding untreated sample set to 1 (not shown). The results are an average of at least three independent experiments performed in triplicate. Error bars represent SD. Statistical significance was determined with a paired two-tailed Student's *t* test using the measurements of the untreated and treated samples as a set of data. *, *P* ≤ 0.01.

The pool of FAs identified by docking prediction was tested for the capacity to affect the VirF-mediated virulence expression in *Shigella*. In particular, we compared the transcriptional levels of the *virF* gene and the VirF-controlled *virB* gene in FA-treated and untreated M90T S. flexneri cells. We also compared the levels of the VirF and VirB proteins under the same conditions. In all assays, the MC-SFAs caproic (CO) and caprylic (CL) acids and the LC-UFA linoleic acid (LI) were included as negative controls since they were not predicted to be efficient interactors. The analysis of the growth curves in the presence of FA concentrations ranging from 0.2% to 0.002% (Fig. S2) shows that, at 0.02%, none of the FAs examined adversely affect the growth of S. flexneri. Therefore, this concentration was used in all subsequent *in vivo* experiments. The results from quantitative reverse transcription PCR (qRT-PCR) assays (Fig. 2A and B) indicate that none of the FAs tested significantly affect the level of *virF* transcription. On the contrary, *virB* transcription is significantly reduced by the presence of most FAs, with MY (LC-SFA), DE (MC-UFAs), and OL (LC-UFA) causing 58%, 64%, and 33% decreases, respectively. The MC-SFAs CA and LA and the LC-UFAs MYO, SA, and PAO lead to an even more impressive reduction (more than 70%). Similar results were also obtained by assessing the transcription of the second *virF*-dependent virulence gene in S. flexneri, *icsA* (Fig. S3). The impact of PA, ST, AR, and the control FAs (CO, CL, and LI) is negligible (Fig. 2A and B).

**FIG 3:**
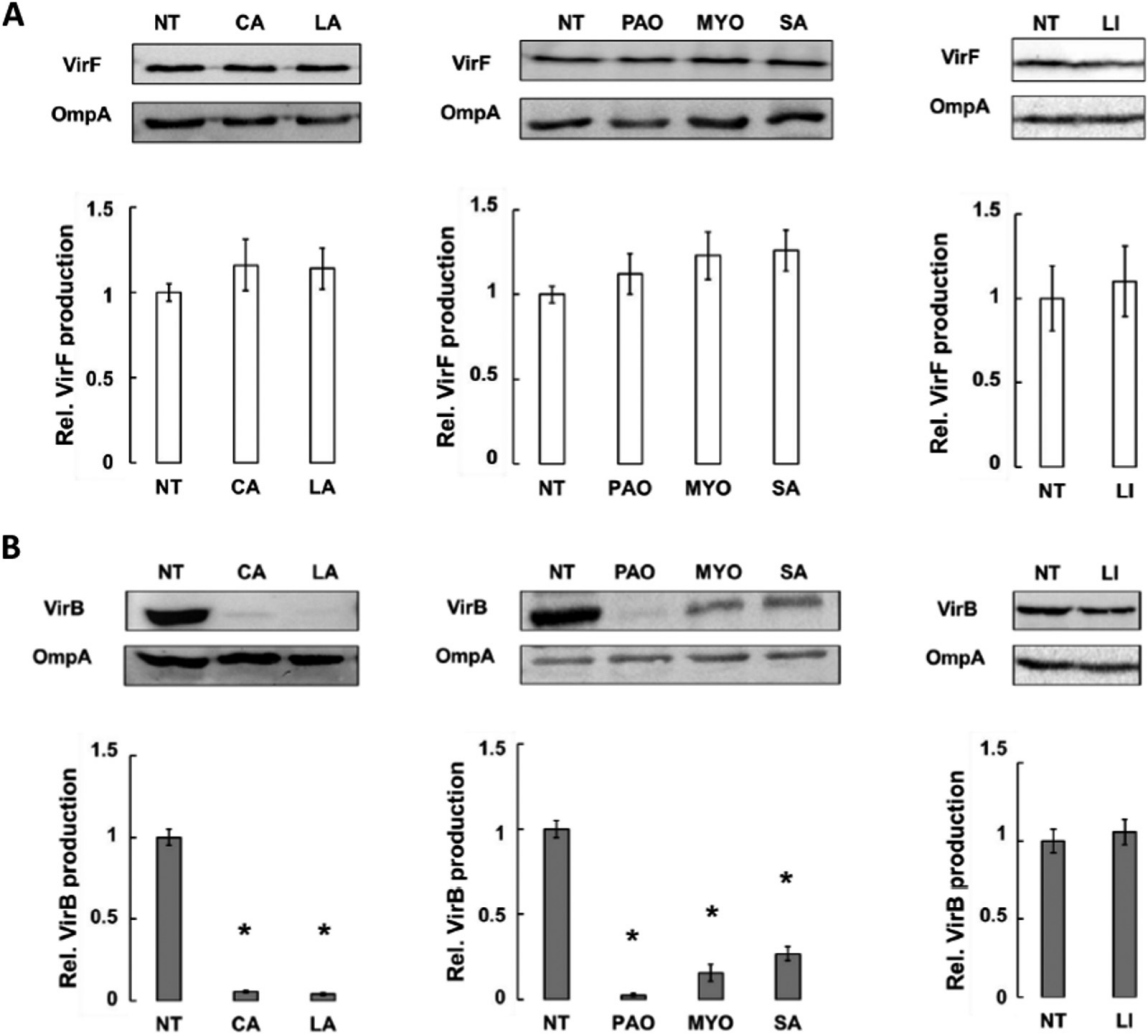
CA and LA, among MC-SFAs, and PAO, MYO, and SA, among LC-UFAs, reduce VirB expression without affecting VirF translation. Western blot (WB) analysis of VirF (A) and VirB (B) proteins in S. flexneri M90T strain grown in the absence or presence of 0.02% CA, LA, PAO, MYO, SA, and LI as a control. Western blot images are representative of three independent experiments (top panels). Densitometric analyses of all experiments are shown under the corresponding WB. Values were obtained by normalizing the VirF and VirB protein levels to those of the OmpA protein and are presented relative to the untreated samples (NT) set to 1. Error bars represent SD. Statistical significance was determined with a paired two-tailed Student's *t* test using the set of measurements of the untreated and treated samples. *, *P* ≤ 0.01.

To rule out that the reduced *virB* transcription depends on the posttranscriptional or translational regulation of *virF*, we measured the levels of the VirF and VirB proteins in S. flexneri cultures treated with CA, LA, PAO, MYO, and SA. To this end, we performed a Western blot assay on total protein extracts from cultures of two S. flexneri M90T-derived strains, M90T *virF*-FT and M90T *virB*-FT, expressing, respectively, a Flag-tagged VirF or VirB protein (14). The results indicate that the VirF (Fig. 3A) and VirB (Fig. 3B) protein levels are consistent with the corresponding transcriptional profiles (Fig. 2A and B), demonstrating that the tested FAs have no relevant effects on VirF. Overall, these data indicate that the CA-, LA-, PAO-, MYO-, and SA-mediated inhibition of *virB* transcription depends on the reduced ability of VirF to activate *virB* transcription.

### FAs bind to the VirF protein and affect its interaction with the *virB* promoter *in vitro*.

**FIG 4:**
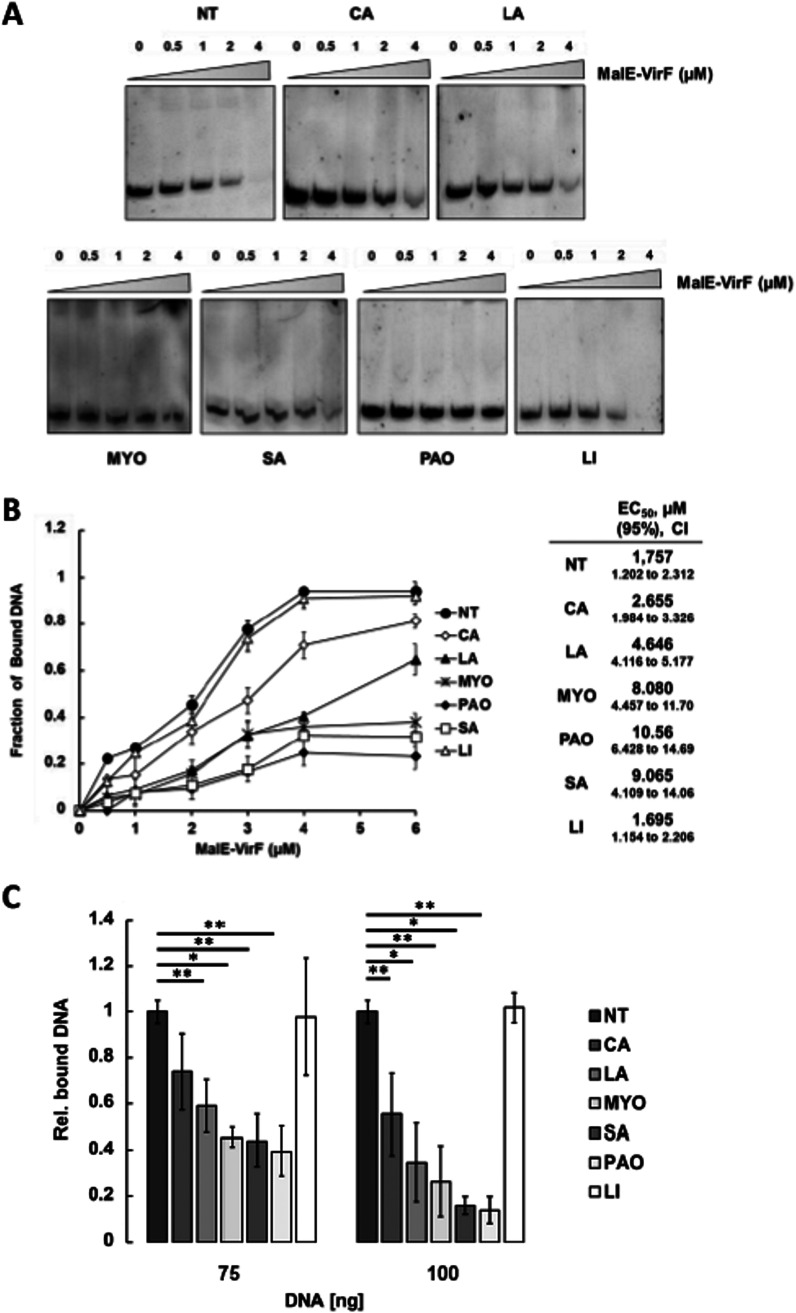
CA and LA, PAO, MYO, and SA decrease the *in vitro* binding affinity of MBP-VirF to the *virB* promoter. MBP-VirF binding to the *virB* promoter was analyzed using EMSA and DPIPA. (A) The EMSA gels shown are representative of three independent experiments. The lanes of all panels consist of 5 μg of DNA fragments with increasing concentrations of MalE-VirF protein. The FAs used for treatment, at a concentration of 0.02%, are indicated above (top panels) and below (bottom panels) each gel. The unbound DNA was evaluated by densitometric scanning of gels from three independent experiments. (B) The average of the values obtained was used to calculate the bound DNA, and the results are shown in the graph. The decreasing binding affinities corresponding to the increase in EC_50_ are shown in the table next to the graph. The 95% confidence intervals (95% CIs) are presented for each affinity in brackets. (C) The results of DPIPA are reported in the bar graph that shows the amount of fluorescence emitted by the P*virB* FITC-DNA retained in the wells by the MalE-VirF protein in the presence of the indicated FAs relative to the corresponding untreated sample. The experiment has been performed using two (75 and 100 ng) saturating concentrations of the P*virB* FITC-DNA fragment, and the results are the average of at least three independent experiments, each performed in triplicate. Error bars represent SD. Statistical significance was determined with a paired two-tailed Student's *t* test using the set of measurements of the untreated and treated samples. **, *P* ≤ 0.05; *, *P* ≤ 0.01.

We asked whether the *virB* inhibitory effect exerted by CA, LA, PAO, MYO, and SA could depend on their direct interaction with VirF, thus hampering its binding to the *virB* promoter. Therefore, we tested these FAs for their binding to purified VirF protein by means of electrophoretic mobility shift assay (EMSA) combining a 194-bp DNA fragment (P*virB*), encompassing the *virB* promoter and regulatory region (from −138 to +57) containing the VirF binding site (−107/−91 and −75/−59) (36, 37), with increasing concentrations (0 to 6 μM) of a purified MalE-VirF fusion protein. LI, previously shown to be ineffective (Fig. 2B), was used as a negative control. The mixtures were run on a polyacrylamide gel to reveal the distribution of VirF-bound and unbound (i.e., free) DNA. The results are presented in Fig. 4. The MalE-VirF-DNA complexes fail to penetrate the gel. This behavior, experienced also by another group (37), could likely depend on the formation of DNA-protein aggregates due to molecular crowding. Nonetheless, the evaluation of the unbound DNA fraction (which migrates regularly throughout the gel) is consistent with the MalE-VirF protein concentration used (Fig. 4A, NT samples). Indeed, plotting the concentration of MalE-VirF versus the fraction of calculated bound P*virB* DNA (Fig. 4B) reveals that for each MalE-VirF concentration, the presence of FAs decreases the amount of bound DNA compared to the FA-untreated samples (NT).

The binding affinity was quantified by determining the half-maximal effective concentration (EC_50_). In the CA-, LA-, MYO-, PAO-, and SA-treated samples, the EC_50_ values (2.665, 4.646, 8.080, 10.560, and 9.050 μM, respectively) are up to 9 (PAO) times higher than in the untreated sample (NT, 1.757 μM) and LI-treated sample (LI, 1.695 μM), with those fatty acids that are the most inhibitory to DNA binding having an EC_50_ of >6 μM (Fig. 4B). To strengthen the results of the EMSAs, we designed a novel assay, the DNA-protein interaction plate assay (DPIPA), based on a modification of the procedure originally proposed to purify MalE fusion proteins (38). Briefly, flat-bottomed wells coated with dextrin as a ligand for affinity adsorption of the MalE-VirF protein were used to study the interaction between VirF and the P*virB* 194-bp 5′-fluorescein isothiocyanate (FITC)-labeled DNA fragment in the presence of the inhibitory FAs (CA, LA, PAO, MYO, and SA). As with the previous experiments, we used LI as a negative control. The results fully confirm the outcome of the EMSAs. Indeed, using saturating amounts of P*virB* (75 and 100 ng), the fold reductions (relative to the untreated samples) of MalE-VirF binding to the DNA are 0.74 and 0.55 with CA, 0.59 and 0.35 with LA, 0.46 and 0.26 with MYO, 0.44 and 0.15 with SA, and 0.39 and 0.14 with PAO, respectively (Fig. 4C). The lack of inhibitory effect of the LI acid (0.97 and 1.10) excludes a nonspecific influence of the tested fatty acids on the VirF-DNA interaction. Altogether, these data demonstrate the interference of CA, LA, PAO, MYO, and SA on the MalE-VirF-DNA interaction and strongly support the hypothesis that these FAs directly interact with VirF, inhibiting its regulatory activity.

### FAs inhibit the *virB* transcription *in vivo* by directly interacting with VirF.

**FIG 5:**
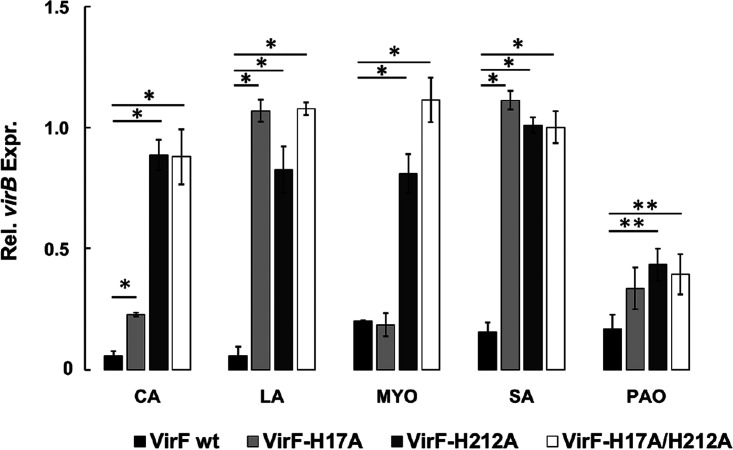
Desensitization profile of VirF H17A, H212A, and H17A-H212A derivatives to CA, LA, PAO, MYO, and SA. The graph shows the transcription of the *virB* gene in the presence of the VirF wild-type protein (black bars) and the H17A (light gray), H212A (dark gray), and H17A-H212A derivatives (white bars), treated with 0.02% CA, LA, PAO, MYO, and SA. The relative values were calculated using, as a calibrator, those obtained for each corresponding untreated sample, set to 1 (not shown). The results are an average of at least three independent experiments performed in triplicate. Error bars represent SD. Statistical significance was determined with a two-tailed Student’s *t* test. **, *P* ≤ 0.05; *, *P* ≤ 0.01.

The *in vivo* and *in vitro* observations presented in the previous sections strongly suggest that FAs have a direct negative impact on the activity of VirF in S. flexneri. The data from our modeling and docking studies predict the VirF residues His17 and His212 to be involved in the interaction with the carboxyl moiety of the FAs. To further verify the interaction of FAs with VirF, we introduced single or double mutations into the VirF protein by replacing the His17 and His212 codons (CAT) with an Ala codon (GCG) in the *virF* gene. The plasmid containing the wild-type *virF* sequence (pMYSH6504) and three mutated derivatives (pVirFH17A, pVirFH212A, and pVirFH17A-H212A) were introduced into the M90T Δ*virF* strain. First, we verified that VirF mutant proteins were expressed equivalently to the wild type by performing a Western blot analysis using the halon anti-VirF antibody (14) (Fig. S4). Then, using the Congo red (CR) plate assay, we showed that the VirF-mutated proteins have a comparable effect on the expression of the virulence system of *Shigella* (39) (Fig. S5). Finally, we used these strains to monitor the effect of the VirF mutations on the transcription of *virB* in the presence of CA, LA, MYO, PAO, and SA, by qRT-PCR analysis (Fig. 5), which reveals that the H212A mutation makes the transcription of *virB* no longer susceptible to the presence of LA, SA, MYO, and CA. In the H17A background, *virB* transcription is insensitive to the presence of LA and SA, while the CA-dependent reduction of *virB* transcription is only barely affected. In strains harboring the H17A or H212A mutations, the transcription of the *virB* gene is partially influenced by PAO. The combination of both mutations does not lead to further reduction of *virB* transcription by PAO. Of note, the comparable level of the change in threshold cycle (Δ*C_T_*) (*C_T_virB*-*C_T_nusA*) values used to assess the VirF protein-dependent *virB* transcriptional profiles excludes a potential misinterpretation of the results due to a possible mutation-dependent inactivation of the VirF protein (Fig. S6). Taken together, these results confirm that the targeted histidine residues are crucial for the direct interaction between FAs and VirF and that this interaction also occurs *in vivo*.

### FAs strongly reduce the S. flexneri infectivity on epithelial cells.

**FIG 6:**
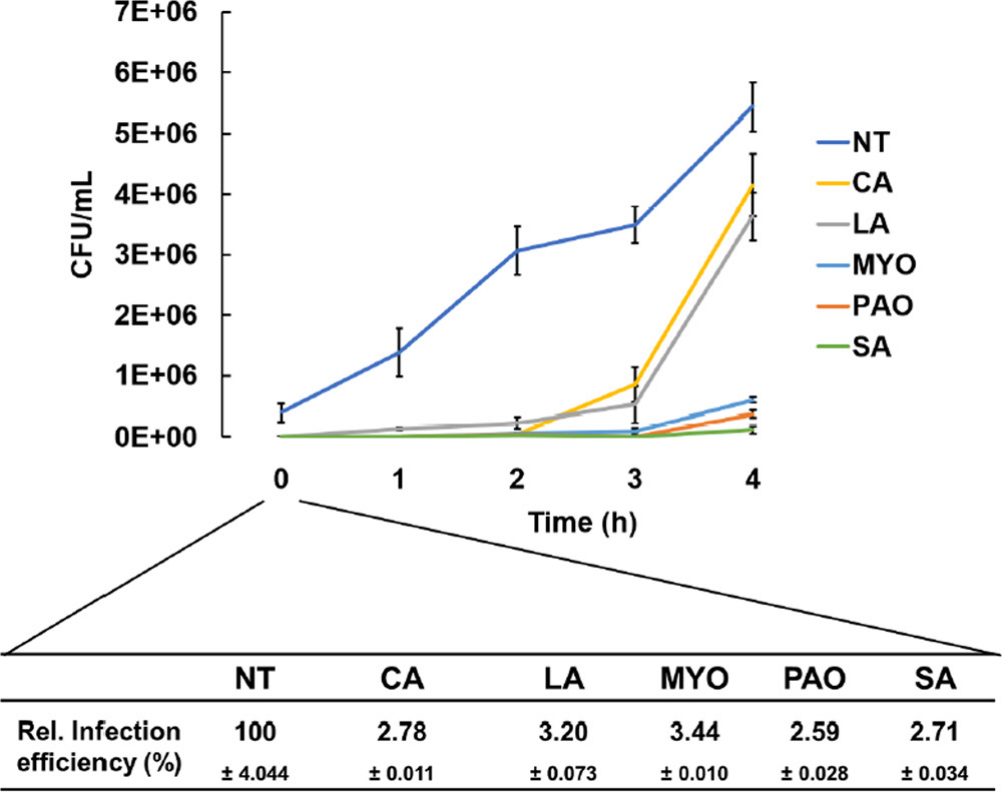
CA, LA, PAO, MYO, and SA impair the S. flexneri infection of Caco-2 epithelial cells. Viable counts (CFU/mL) of intracellular bacteria over 4 h of infection (0 to 4). The infection was carried out at an MOI of 100 with S. flexneri M90T grown in the absence (NT) or with 0.02% CA, LA, MYO, SA, and PAO. The results are the average of at least three independent experiments. The infection efficiency (0 h), compared with the untreated sample, is shown below the graph. The statistical significance was determined by the two-way analysis of variance (ANOVA) (*P* = 0.0015). Error bars represent the SD.

To test whether the inactivation of VirF by FAs has a direct impact on the virulence phenotype of *Shigella*, we analyzed the capability of the FA-treated S. flexneri M90T strain to invade epithelial cells and proliferate within them. Caco-2 cells were infected with M90T previously grown in the presence of CA, LA, MYO, PAO, and SA. The infection was monitored for 4 h by lysing the epithelial cells and measuring intracellular bacteria by viable counts. The number of intracellular bacteria recovered at time zero was used to calculate the efficiency of invasion. We observe a strong inhibition of invasiveness following FA treatment (Fig. 6). Indeed, the percentages of viable bacteria from CA-, LA-, MYO-, PAO-, and SA-pretreated samples are 2.78, 3.20, 3.44, 2.59, and 2.71, respectively. The analysis of subsequent infection time points (1 to 4 h) shows that in the absence of bacterial pretreatment with FAs, the number of viable bacteria recovered from the epithelial cells increases almost linearly over the 4 h postinfection (hpi) period (Fig. 6). In contrast, when M90T is grown in the presence of CA or LA, the number of intracellular bacteria remains almost unchanged during the first 2 hpi and shows a slight increase after 3 hpi and a substantial increase after 4 hpi. Pretreatment of bacteria with MYO, PAO, and SA results in a more stable inhibition as indicated by the fact that the number of viable bacteria recovered remains unchanged over the first 3 hpi and shows only a slight increase at 4 hpi. These results demonstrate that VirF inhibition mediated by the FAs tested hinders the expression of the S. flexneri virulence system, adversely affecting bacterial entry into and proliferation within host cells.

## DISCUSSION

In both prokaryotes and eukaryotes, fatty acids (FAs) are involved in a wide range of biological processes, ranging from modulation of membrane properties to complex processes such as differentiation, proliferation, secretion, and invasion (40). In bacteria, FAs are also known to induce cell death and inhibit growth by acting on multiple cellular targets (41). When present, at concentrations below the MIC, FAs have been reported to affect biofilm formation as well as the full expression of the virulence phenotype in several pathogenic microorganisms (42). The antivirulence activity of FAs has been well characterized in Candida albicans (43), Acinetobacter baumannii (44), Xanthomonas citri subsp. *citri* (45), Yersinia enterocolitica (46), enterotoxigenic Escherichia coli (ETEC) (31), and Vibrio cholerae (30). In particular, in ETEC and V. cholerae, the antivirulence effect of FAs is mediated by the direct interaction of FAs with the transcriptional activators of the AraC-XylS family Rns and ToxT (31, 32) In the present study, we demonstrate that in *Shigella*, some FAs negatively affect virulence by directly interacting with the VirF protein, an AraC-XylS protein responsible for triggering the *Shigella* invasive program (15).

We first compared the structure of the *Shigella* VirF protein, obtained by homology modeling and AlphaFold2, with the crystal structure of ETEC Rns and V. cholerae ToxT, two proteins that belong to the same subgroup of AraC-XylS virulence regulators and whose activity is inhibited by specific FAs (28, 31). We observe that, as in Rns and ToxT, also in the VirF, the companion domain (CD) folds in a typical jelly roll module with a hydrophobic pocket that can accommodate small molecules (Fig. 1).

The interaction of the Rns CD with decanoic acid abolishes the Rns-dependent transcription of the ETEC genes encoding colonization factors (27, 31), and some unsaturated fatty acids inhibit the dimerization of V. cholerae ToxT, hampering the transcription of operons critical for intestinal colonization (28–30). Based on these observations, we performed a molecular docking analysis, which allowed us to identify a pool of FAs (Table 1) potentially able to bind the VirF CD. These FAs were then experimentally tested to assess their impact on the VirF-mediated transcription of the *virB* gene. Transcriptional and translational *in vivo* analyses (Fig. 2A and B) show that the presence of CA and LA (MC-SFAs) or MYO, PAO, and SA (LC-UFAs) causes a strong reduction of *virB* transcription without affecting the level of VirF. To verify whether these results depend on a direct interaction between the selected FAs and VirF protein, as suggested by the molecular docking predictions, we performed two different *in vitro* assays (EMSA and DPIPA). The results confirm that all selected FAs bind to the VirF protein and limit its ability to interact with the *virB* promoter (Fig. 4A to C), with the LC-UFAs SA, MYO, and PAO being more effective than the MC-SFAs CA and LA. The higher *in vivo* effectiveness of CA and LA in reducing the VirF-mediated transcription of *virB*, compared to SA and MYO (Fig. 2B), can be traced to differences between the LC-UFAs’ and MC-SFAs’ membrane transport and diffusion mechanisms and their metabolic fates (47, 48).

**FIG 7:**
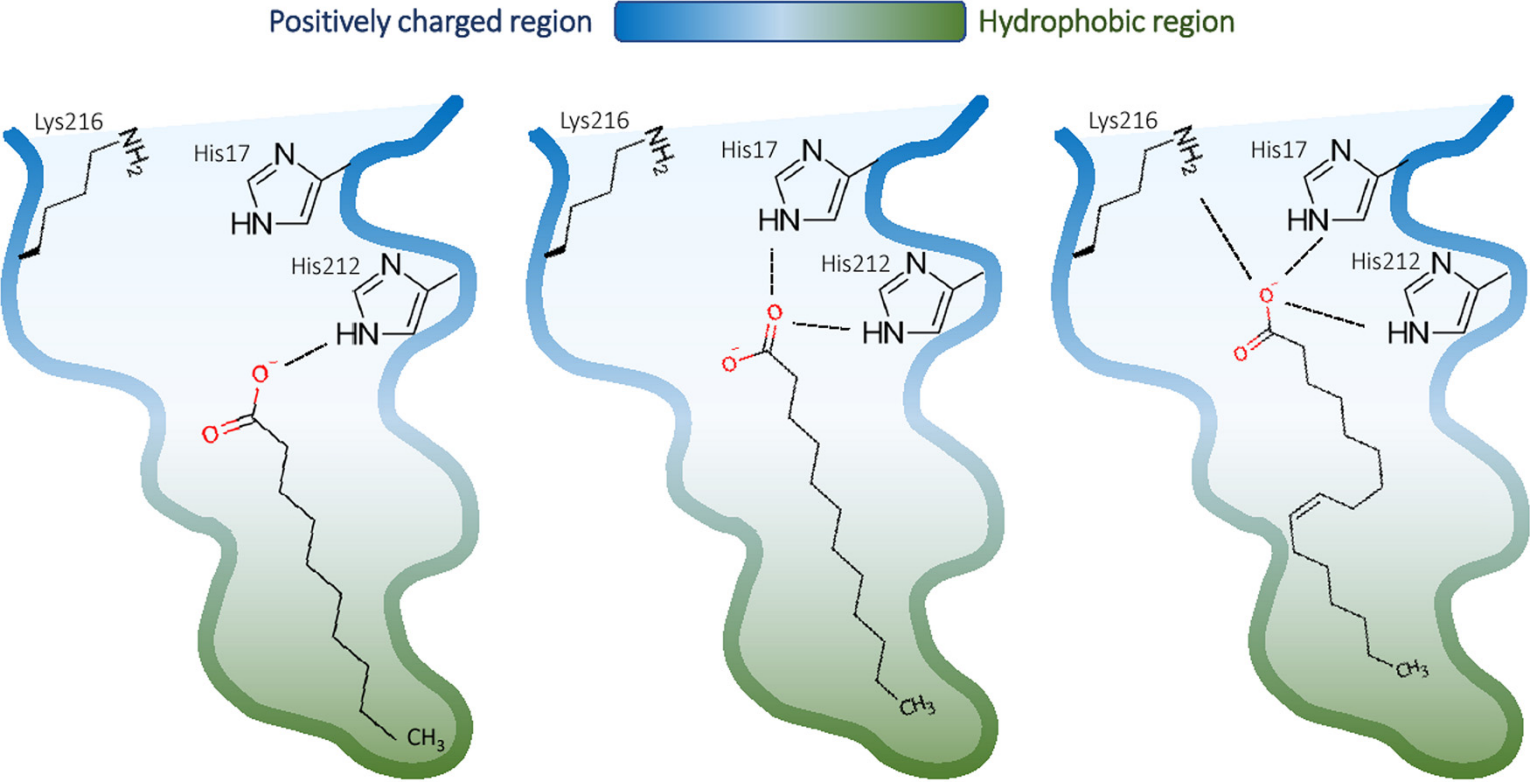
Proposed model of interaction of FAs with VirF. The binding cleft of VirF is shown colored by charge (blue) and hydrophobicity (green). The schematic positions of CA (left), LA (middle), and PAO (right) FAs are shown, together with residues possibly involved in the interaction.

Our docking analysis predicts two specific histidine residues (His17 and His212) of VirF to interact with the carboxyl group of the selected FAs (Fig. 1). Transcription experiments *in vivo* (Fig. 5) show that, overall, the replacement of these histidine residues with alanine (VirF H17A and H212A) restores the capacity of VirF to activate the *virB* promoter in the presence of the selected FAs. In particular, VirF H17A and H212A have a different “desensitization” effectiveness (Fig. 5), suggesting that the His212 residue of VirF is required for the interaction with CA and MYO and that the presence of both His212 and His17 residues is essential for the interaction with LA and SA but only partially contributes to the interaction with PAO. According to the docking analysis, CA adopts an extended conformation within the VirF binding cleft, preferring the interaction with His212, which is placed deeper inside the cleft than His17 (Fig. 7). The same happens for MYO, which has a longer aliphatic chain than CA but, due to the C9-C10 double bond and the “L-shaped” conformation within the cleft, occupies the same volume as CA. As for PAO, according to the different poses found in the docking analysis, it may adopt alternative conformations within the cleft, being able to reach also other polar residues lining the cleft (e.g., K216) (Fig. 7). A more in-depth structural analysis of the VirF protein will allow understanding of why both histidine residues of VirF are required for the functional interaction with SA or LA.

The capability of *Shigella* to invade host cells and proliferate inside them is strictly related to the expression of a specific VirF-regulated virulence system (49). The effect of the selected FAs has been analyzed by assessing the number of intracellular bacteria in Caco-2 cells. We observe that pretreatment of S. flexneri cultures with CA, LA, MYO, SA, and PAO has a strong impact on bacterial entry as well as on the proliferation of intracellular bacteria (Fig. 6). In particular, pretreatments with MYO, SA, or PAO produce a more durable repressive effect, well in line with the higher stability of the interaction between VirF and these FAs (Fig. 4A to C). We speculate that within the host cells, in the absence of free fatty acids, the VirF protein releases CA and LA more easily than MYO, SA, and PAO, thus allowing the virulence system to reactivate more readily.

Altogether, our results suggest that VirF, as previously proposed for ToxT and Rns (28, 31), once bound by the selected FAs, undergoes a structural transition from an “open” to a “closed” conformation, becoming unable to interact with the *virB* promoter. This mechanism could be responsible for the programmed control of *Shigella* virulence expression during its journey through the human intestine toward the colonic mucosa. The distribution of long- and medium-chain fatty acids and their bioavailability in the gut have not yet been fully elucidated; however, it is reasonable to assume that the concentration of FAs, derived from bile and diet, is low in the large intestine, as they are absorbed along the small intestine (34). Moreover, it has been proposed that the concentration of FAs may decrease further within the mucosal layer (29). Therefore, considering that human bile is estimated to contain approximately 2% PAO (50), which we have shown to be the most effective *Shigella* virulence inhibitor among those we tested, we hypothesize that virulence expression is repressed by the presence of this FA, or of other dietary-derived inhibitory FAs, in the small intestine, thus allowing *Shigella* to restore its virulent state once it arrives in the large intestine and nears the intestinal mucosa.

*Shigella* species are a serious threat to public health, as they are a major cause of diarrheal deaths worldwide (51, 52). The absence of an effective vaccine against *Shigella* spp. currently requires the use of antibiotics as a prominent clinical approach (53). This has resulted in an alarming increase in antibiotic-resistant *Shigella* strains isolated in recent years (54). A viable alternative, or at least a complementary approach, to antibiotic therapy is represented by drugs able to target bacterial virulence, rather than bacterial growth, thus mitigating the emergence of antibiotic-resistant strains. Given its central position in the *Shigella* virulence system, VirF can be considered an ideal antivirulence target (17, 55). This is confirmed by research on promising compounds, such as 19615 (methyl-[2-(2-phenyl-4a,9b-dihydro-benzo[4,5]furo[3,2-d]pyrimidin-4-y1oxy)-ethyl]-amine) (56) and SE-1 (1-butyl-4-nitromethyl-3-quinolin-2-yl-4H-quinoline]) (57), which can interact with the DNA binding domain of VirF and thus repress its binding to the *virB* promoter.

VirF is inhibited by LC-UFAs and MC-SFAs, as in the case of the FA-mediated inhibition of ToxT in Vibrio cholerae, Rns in enterohemorrhagic E. coli (ETEC) and RtsA, HilC, and HilD in S. enterica (29, 31–33). Since VirF, Rns, RtsA, HilC, and HilD share high homology with other virulence regulators of the AraC-XylS family, such as AggR in enteroaggregative E. coli (EAEC), CsvR, FapR, and CfaD in enterotoxigenic E. coli (ETEC), RegA in Citrobacter rodentium, RegR in rabbit-specific enteropathogenic E. coli (REPEC), and PerA in enteropathogenic E. coli (EPEC) (26), antivirulence drugs originally selected for single pathogen may show a broader spectrum of activity than expected.

The posttranslational FA-dependent negative VirF regulation shown in this paper provides new insight into the structure and function of this regulator and its interaction with inhibitory ligands, and it may represent a new opportunity to rationally design molecules that can act as antivirulence drugs in the treatment of shigellosis. The recent discovery of such antivirulence molecules, capable of mimicking the binding of inhibitory FAs in ToxT (58–60), supports this view.

## MATERIALS AND METHODS

### Computational methods.

The three-dimensional model of VirF was obtained using, as a template, the homologous transcriptional regulator Rns (https://www.rcsb.org/; PDB accession no. 6XIU; percentage of identity, 41%) (31). Initial modeling with AlphaFold2 (version 2.0.1) (61) and validation permitted us to obtain a final model whose metrics (Ramachandran outliers, energy plots, etc.) were close to a low-resolution crystal structure (data not shown). The docking of FAs into the active site was performed using the Molegro Virtual Docker (MVD) software v7.0 (Molexus). The three-dimensional (3D) structure of VirF was prepared by adding bond orders, hybridization, explicit hydrogens, charges, and Tripos atom types. A search space with a 15.0-Å radius centered on the binding site cleft was used for docking, and the MolDock score with a grid resolution of 0.20 Å was used as the scoring function. One hundred runs were defined, and the retrieved poses were ranked according to the rerank score in MVD, which correlates with protein-ligand affinity and dissociation constants (62). Compounds were also docked using AutoDock v4.2.5.1 (63), with the Lamarckian genetic algorithm and a population of 150 individuals. Poses showing a similar conformation (root mean square deviation [RMSD] of < 2.0 Å), as assessed by MVD and AutoDock, were kept, and the first-ranking one was chosen for further analysis.

### Bacterial strains and general growth conditions.

The bacterial strains used in this study are listed in Table S1 in the supplemental material. Bacterial cells were grown aerobically in Luria-Bertani (LB) medium (Sigma-Aldrich) at 37°C, and when requested, antibiotics were added at the following final concentrations: 100 μg/mL ampicillin, 30 μg/mL kanamycin, and 10 μg/mL streptomycin. The Congo red (CR) plate assay was carried out using CR (Sigma-Aldrich) supplemented with 0.01% tryptic soy agar (TSA; Becton, Dickinson GmbH) plates. FAs used in this work include caproic acid (CAS no. 142-62-1), caprylic acid (CAS no. 124-07-2), capric acid (CAS no. 334-48-5), lauric acid (CAS no. 143-07-7), myristic acid (CAS no. 544-63-8), palmitic acid (CAS no. 57-10-3), stearic acid (CAS no. 57-11-4), arachidic acid (CAS no. 506-30-9), decenoic acid (CAS no. 14436-32-9), palmitoleic acid (CAS no. 373-49-9), oleic acid (CAS no. 112-80-1), and linoleic acid (CAS no. 60-33-3; all from Sigma-Aldrich), myristoleic acid (CAS no. 544-64-9; A2Bchem), and sapienic acid (CAS no. 17004-51-2; Santa Cruz Biotechnologies). To evaluate the effect of FAs on bacterial growth and gene expression, FAs were dissolved in methanol and used at the indicated final concentrations in the LB medium.

### DNA methods.

DNA purification, plasmid transformation, and gel electrophoresis were performed as previously described (64). The oligonucleotide sequences, designed based on the M90T genome, are reported in Table S2. PCRs were routinely performed using the DreamTaq DNA polymerase (Thermo Fisher Scientific) or, when required, a higher fidelity of PCR product, the Ex Taq DNA polymerase (TaKaRa). Plasmids used in this work are listed in Table S3. Plasmids pVirF-H17A, pVirF-H212A, and pVirF-H17A-H212A were obtained by site-directed mutagenesis using the GeneArt site-directed mutagenesis system (Thermo Fisher Scientific) with pMYSH6504 (Table S3) as the template and oligonucleotides p6504_H17A F/p6504_H17A R or p6504_H212A F/p6504_H212A R. The pMALcF1 construct was realized by cloning the BamHI-restricted amplicon, obtained using the CF4B-CE24 oligonucleotide pair and pMYSH6504 as a template, into the BamHI site of pMALc2x (New England Biolabs Inc.). All constructs were confirmed by DNA sequence analysis. The M90T VirB-FT strain was obtained using the one-step method of gene inactivation (65) by transforming M90T pKD46 with an amplicon obtained with the pair VirBFT F-VirBFT R and pSUB11 as a template.

### RNA isolation and quantitative reverse transcritpion PCR.

Bacterial RNA purification was performed as previously described (66), and cDNA synthesis was obtained with the high-capacity cDNA reverse transcription kit (Applied Biosystems). Briefly, 1.5 μg of total RNA from bacteria was treated with DNase I and then retrotranscribed in a 20-μL reaction mix following the manufacturer’s instructions.

qRT-PCR analysis was performed on a StepOnePlus real-time PCR system (Applied Biosystems). The reaction volume was 20 μL containing Power SYBR green PCR master mix (Applied Biosystems), 2 μL of cDNA sample, and oligonucleotides specific for the *virF*, *virB*, *icsA* and *nusA* genes (300 nM each). The cycling conditions were as follows: 1 cycle at 95°C for 2 min and 40 cycles at 95°C for 10 s, followed by 60°C for 30 s. The quantitative analysis of the transcripts was based on the 2^−ΔΔ^*^CT^* method (67), and the results are indicated as “relative expression” to the reference sample. All the primer pairs used for qRT-PCR, designed using the Primer Express software v2.0, are reported in Table S2 and were experimentally validated.

### SDS-PAGE and immunoblot analysis.

Bacteria pellets were resuspended in phosphate-buffered saline (PBS) with 1× final sample buffer (FSB) and, after boiling at 100°C, loaded on 12.5% SDS-PAGE. A protein molecular weight marker (page ruler; Thermo Fisher) was included in each electrophoresis run. Proteins were transferred onto nitrocellulose membranes (Hybond-P; Millipore) and filter incubated with mouse monoclonal anti-FLAG M2 antibody (Sigma-Aldrich), rabbit polyclonal anti-OmpA, or anti-VirF halon (68) antibodies. Secondary antibodies used were horseradish peroxidase (HRP)-conjugated goat anti-mouse and anti-rabbit IgG (Sigma-Aldrich). Signals were produced with ECL Star (Euroclone) and detected with ChemiDoc gel imaging system (Bio-Rad Laboratories). The densitometric analysis was performed by ImageJ software.

### Cell cultures and infections.

Infection experiments were performed by using Caco-2 cell lines. Human Caco-2 epithelial cells (American Type Culture Collection, Manassas, VA) were grown at 37°C in a humidified 5% CO_2_ atmosphere in DF10, which consists of Dulbecco modified essential medium (DMEM; Gibco) containing 10% heat-inactivated fetal bovine serum (FBS; Euroclone), 2 mM l-glutamine, 0.05 IU/mL penicillin, and 0.05 IU/mL streptomycin (PS), as previously described. For bacterial infection (69), cells were seeded in 6-well tissue culture plates (Falcon) at a density of 8 × 10^5^ cells/well in DF10. After 24 h, cells were serum starved overnight in DMEM supplemented with 0.5% FBS and PS (DF0.5). Two hours before bacterial infection, DF0.5 was replaced with fresh DMEM containing only l-glutamine. Untreated and 0.02% FA-treated bacteria were added to Caco-2 cells at a multiplicity of infection (MOI) of 100. Plates were centrifuged for 15 min at 750 × *g* and incubated for 45 min at 37°C under a 5% CO_2_ atmosphere to allow bacterial entry. Afterward, extracellular bacteria were removed by complete washing with PBS. This point was considered time zero (0). Fresh DMEM medium containing gentamicin (100 μg/mL) was added to each plate to kill extracellular bacteria, and the infected cells were incubated at 37°C for up to 4 h. To calculate the number of intracellular bacteria at each infection times point, cells were lysed with 1% Triton X-100, bacteria were recovered and washed with 0.9% NaCl, and serial dilutions were plated on LB agar. Bacterial proliferation was analyzed by calculating the CFU per milliliter obtained over 4 hpi (0 to 4). The infection efficiencies were calculated as the percentages of bacteria that penetrated Caco-2 epithelial cells at time zero relative to the preinfection control.

### Purification of MalE-VirF.

Overnight cultures of E. coli XL1BLUE pMALcF1 were diluted 100-fold in 1 L LB medium containing 0.2% glucose and incubated at 37°C. When the culture density reached an optical density at 600 nm (OD_600_) of 0.5, 0.3 mM IPTG (isopropyl-β-d-thiogalactopyranoside) was added, and incubation was continued for a further 4 h at 37°C. Cells harvested by centrifugation were resuspended in 10 mL of buffer A (20 mM Tris-HCl, pH 7.5, 1 mM EDTA, and 200 mM NaCl). Bacterial cells were then disrupted by sonication on ice and centrifuged at 12,000 rpm at 4°C for 20 min to remove cell debris. The supernatant was diluted 5-fold with buffer A and loaded onto a 5-mL column of amylose resin (New England Biolabs) preequilibrated in buffer A at a flow rate of 1 mL/min. The column was washed with 40 mL of buffer A, and the fusion protein was eluted with 20 mL of buffer A containing 10 mM maltose. Fractions containing MalE-VirF, as judged by SDS-PAGE analysis, were pooled. Then, through ultrafiltration cycles performed with 50-kDa-molecular-weight-cutoff (MWCO) (Millipore) filter devices, protein fractions were washed with buffer A (to remove maltose) and finally concentrated in the same buffer. Protein concentration was calculated using a theoretical extinction coefficient at 280 nm of 89,730 M^−1 ^cm^−1^ (calculated with the Expasy ProtParam tool).

### EMSAs.

Electrophoretic mobility shift assays (EMSAs) were performed using the MalE-VirF protein and a DNA fragment as a probe, obtained by PCR using the oligonucleotide pair pvirB_F-pvirB_R and the plasmid pBN1 as a template. The 194-bp *PvirB* amplicon includes the promoter-proximal VirF binding site of the *virB* regulatory region (37). DNA probe (5 ng) was incubated without or with increasing concentration of purified MalE-VirF (0 to 6 μM) in 10 μL binding buffer (50 mM Tris-HCl, pH 7.5, 0.5 mM dithiothreitol [DTT], 25 mM NaCl, and 0.05% NP-40) at room temperature for 20 min. When required, 0.02% (vol/vol) of each FA was added. Samples were run in native 6% polyacrylamide gels (29:1 ratio of acrylamide to bisacrylamide) at room temperature at 70 V for 50 min in 0.25× Tris-borate-EDTA (TBE), pH 9.5. Gels were stained with SYBR green (Sigma-Aldrich) in 15 mL of 0.5× TBE and visualized on a UV transilluminator. Densitometric measurements of free DNA bands were converted into percentage of relative free DNA in the absence of binding protein. Half-maximal effective concentration (EC_50_) of DNA-protein binding equilibria was obtained from the least square fitting of data to the Hill equation and expressed as the average ± standard deviation of values obtained in three EMSAs.

### DPIPA.

The DNA Protein Interaction Plate Assay (DPIPA) utilizes the dextrin-coated flat-bottom polystyrene microplate (Nunc Maxisorp; Thermo Fisher Scientific) as solid matrix for affinity adsorption of the MalE-VirF protein. This was then used to measure the amount of the P*virB* DNA fragment comprising the proximal VirF binding site on the *virB* promoter (37) and obtained by PCR amplification using the 5′-fluorescein isothiocyanate (FITC)-labeled oligonucleotide pair, pvirB_F FITC-pvirB_R FITC (Table S2) and the pBN1 plasmid as the template. Briefly, the 96-well plate was treated with 150 μL of 0.5% (wt/vol) dextrin from maize starch (Sigma-Aldrich) dissolved in 100 mM sodium phosphate buffer, pH 7 (PBS), overnight at 4°C in a humid chamber. Wells were then blocked for 1 h with 150 μL of 1.5% bovine serum albumin (BSA) in PBS at 37°C. After washing with PBS, 50 μL of binding buffer solution (100 mM Tris-HCl, pH 7.5, 1 mM DTT, 50 mM NaCl, and 0.1% NP-40) containing 1 μM MalE-VirF protein, 75 or 100 ng of FITC-labeled PCR DNA fragments, and, when requested, 0.02% FAs was added to the wells and incubated at room temperature for 30 min with gentle agitation. Finally, the supernatant was removed, and the fluorescence of bound DNA was detected with the CLARIOStar microplate reader (BMG Labtech, Offenburg, Germany). The concentration ratio between dextrin and MalE-VirF used was established by evaluating the combination of different concentrations of dextrin (0% to 1%, wt/vol) with increasing concentrations of MalE-VirF protein (0.5 μM to 4 μM) and estimating the amount of bound MalE-VirF by evaluating the unbound protein by SDS-PAGE gel electrophoresis. Furthermore, we excluded FA interference in dextrin-MalE-VirF binding, as no differences in the amount of unbound MalE-VirF protein were detected in the untreated versus the FA-treated samples. Finally, the amount of DNA to be used in the assay was defined based on the saturation point. This was assessed by adding an increasing concentration of FITC-labeled PCR DNA fragments (from 5 ng to 100 ng) to the dextrin-MalE-VirF complex (Fig. S7).

### Data availability.

The data sets used and/or analyzed during the current study are available from the corresponding author upon reasonable request.
