# Supplementary material for: Fatty Acids Abolish Shigella Virulence by Inhibiting Its Master Regulator, VirF
Source: Microbiol Spectr. 2023 May 4;11(3):e00778-23. doi: 10.1128/spectrum.00778-23 (PMC10269687; doi:10.1128/spectrum.00778-23)
Supplement: Supplemental file 1 — Supplemental material. Download spectrum.00778-23-s0001.pdf, PDF file, 1.1 MB [file spectrum.00778-23-s0001.pdf]

Supplemental material

Tables S1

Strains used in this work

Strain	Characteristic	Source/References
XL1BLUE	*recA1 endA1 gyrA96 thi-1 hsdR17 supE44 relA1 lac [F proAB lacIqZΔM15 Tn10 (Tetr)]*	Agilent Technologies
M90T	*S.flexeneri* 5a; wild type harbouring 220-kb invasion plasmid pWR 100	(1)
M90T F-FT	M90T with VirF-tagged (3xFLAG) protein	(2)
M90T B-FT	M90T with VirB-tagged (3xFLAG) protein	This study
M90T Fd	M90T with *virF* gene deletion (Δ *virF*)	(3)

Table S2

Oligos used in this work

Name	5’-3’ sequence
VirBFT F	CTATTCGGGATATAATTTCTCGCCATCTATCGTCTTCAACTACAAAGACCATGACGG
VirBFT R	TCAGCTGAATATTTTCGGCCAGTCACTCATCTTCACATATGAATATCCTCCTTAG
p6504_H17A F	GATATAAAGGTTCGCTTGGCGAACTATATTATTTTATATG
p6504_H17A R	CATATAAAATAATATAGTTCGCCAAGCGAACCTTTATATC
p6504_H212A F	CTTGATATTCGCATGGCGCATGCAGCAAAG
p6504_H212A R	AAGCTTTGCTGCATGCGCCATGCGAATATC
qvirF F	AAAGGTGTTCAATGACGGTTAGC
qvirF R	CAATTTGCCCTTCATCGATAGTC
qvirB F	GGAAGGCCAAAAGAAAGAGTTTACA
qvirB R	GAGGAATCTTGGCTTTGATAAAGG
pvirB_F	CTCACATCAGAGCTCCA
pvirB R	ATCACACCCTGTTTATTCATATTG
qnusA F	TGAAGCCGCACGTTATGAAG
qnusA R	TCAACGTAATCGCCCAAGTT
qicsA F	TGATGGACTTTCTCCCTTGGG
qicsA R	TACCACGCATCCATTCCATCT
pvirB_F FITC	5’FITC - GGAGCTCTCACATCAGAGCTCCAC
pvirB R FITC	5’FITC - CCATATCACACCCTGTTTATTCATATTG

Table S3

Plasmid used in this work

Plasmids	Characteristic	Source/References
pMALc2x	Expression Vector	New England Biolabs
pMALcF1	pMALc2x derivatives containing the *virF* gene	This study
pKD46	Temperature-sensitive replicon that carried bacteriophage λ red gene (β, γ and exo) under control of inducible ParaBAD promoter	(4)
pSUB11	Plasmid carrying 3xFLAG epitope, template for PCR	(5)
pBN1	pBR322-derived vector carrying the *virB* gene	(6)
pMYSH6504	pBR322 derivative carrying the *Shigella virF* gene	(7)
pVirFH17A	pMYSH6504 derivative carrying the*virF* mutated gene (cat -> gcg; +110 +112)	This study
pVirFH212A	pMYSH6504 derivative carrying the*virF* mutated gene (cat -> gcg; +695 +697)	This study
pVirFH17A/H212A	pMYSH6504 derivative carrying the *virF* mutated gene (cat -> gcg +110 +112 and cat -> gcg +695 +697)	This study

Fig. S1

Dope Score Plot, Ramachandran Plot and AlphaFold (AF) model confidence of the VirF model (8)

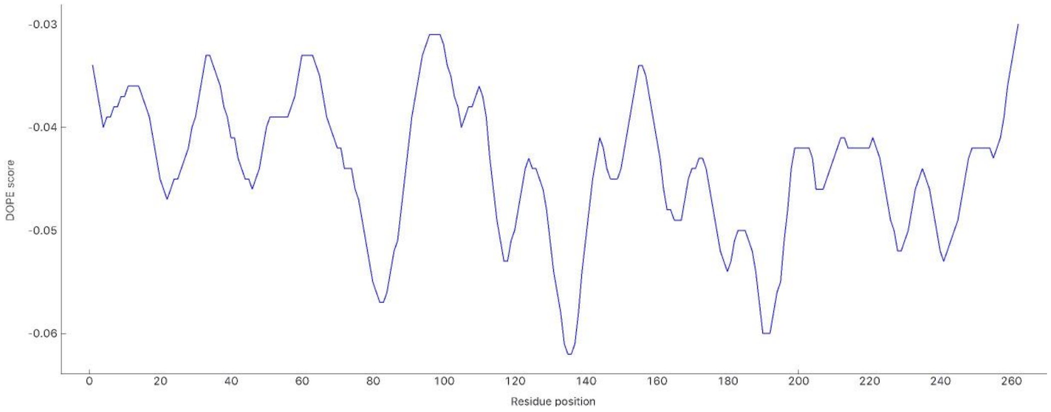

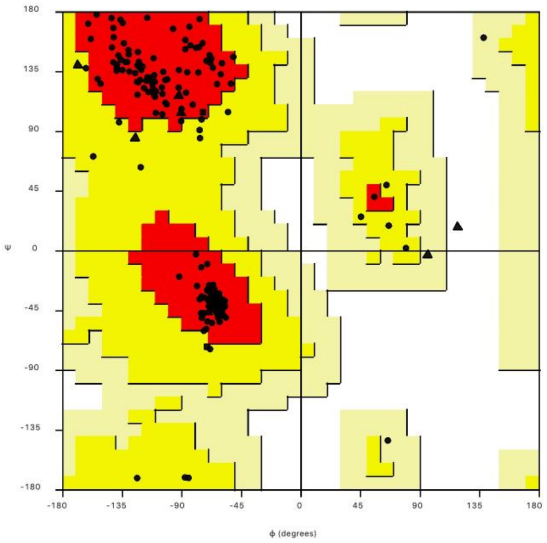

Residues in the most favored regions:
229 (92.3%)

Residues in additionally allowed regions:
17 (92.3%)

Residues in generously allowed regions:
2 (0.8%)

Residues in disallowed regions:
0 (0.0%)

AF Model Confidence:

■	Very high	(pLDDT > 90)
■	Confident	(90 > pLDDT > 70)
■	Low	(70 > pLDDT > 50)
■	Very low	(pLDDT < 50)

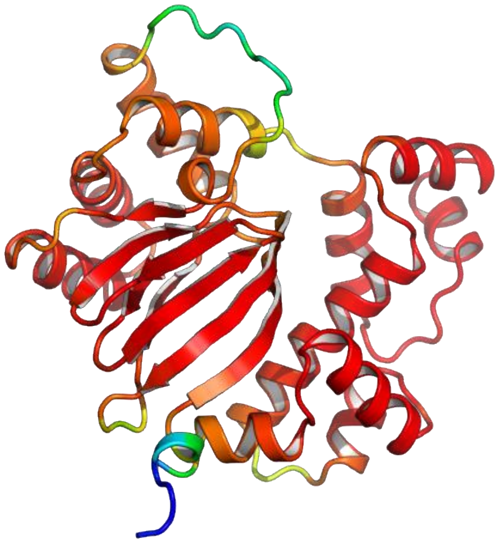

Fig. S2

M90T growth curves in presence of increasing concentration of FA

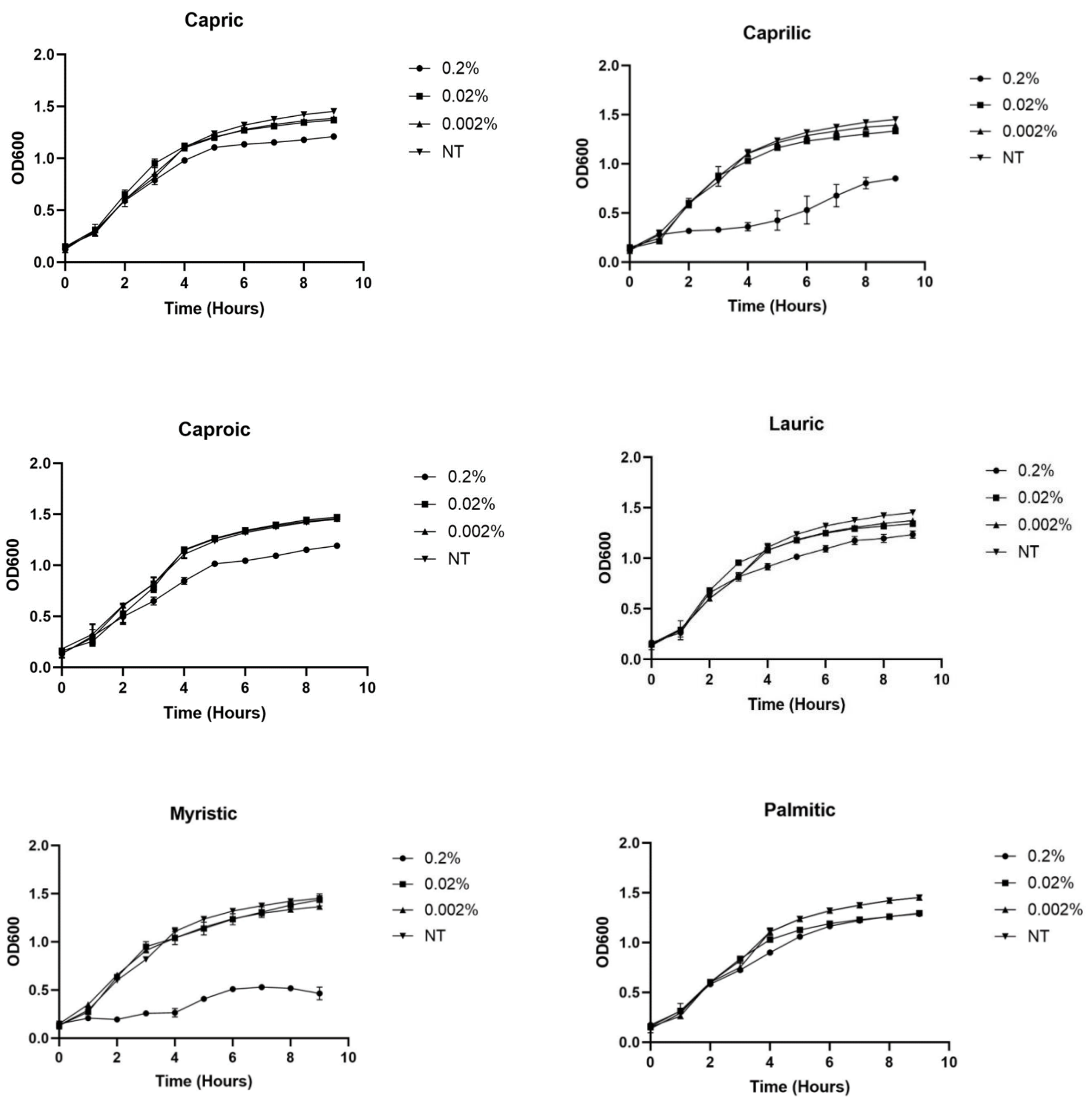

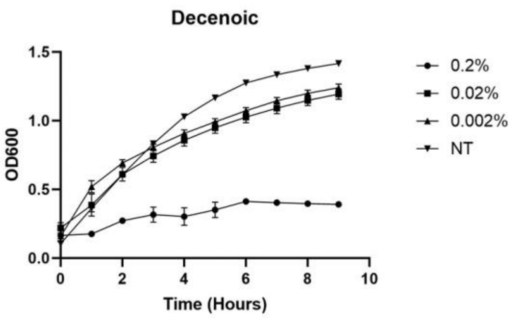
Decenoic
OD600
Time (Hours)
0.2%
0.02%
0.002%
NT

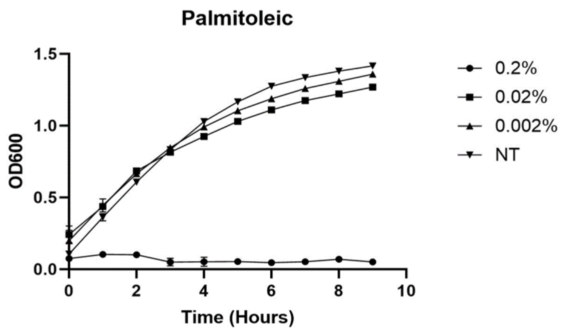
Palmitoleic
OD600
Time (Hours)
0.2%
0.02%
0.002%
NT

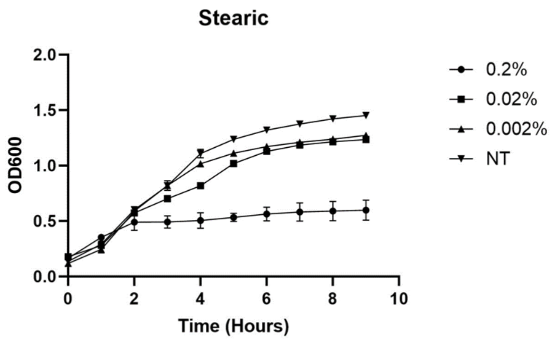
Stearic
OD600
Time (Hours)
0.2%
0.02%
0.002%
NT

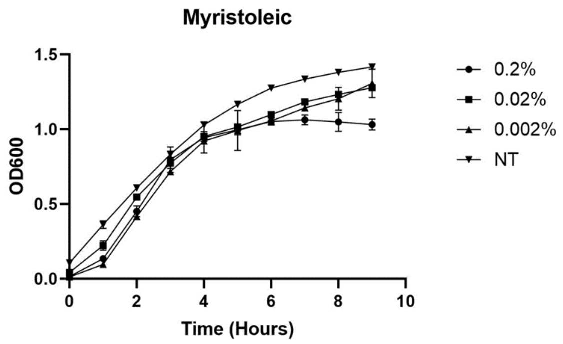
Myristoleic
OD600
Time (Hours)
0.2%
0.02%
0.002%
NT

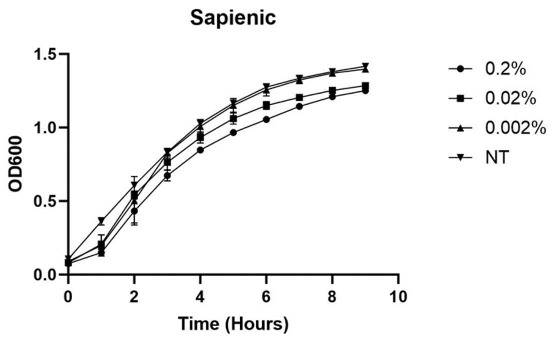
Sapienic
OD600
Time (Hours)
0.2%
0.02%
0.002%
NT

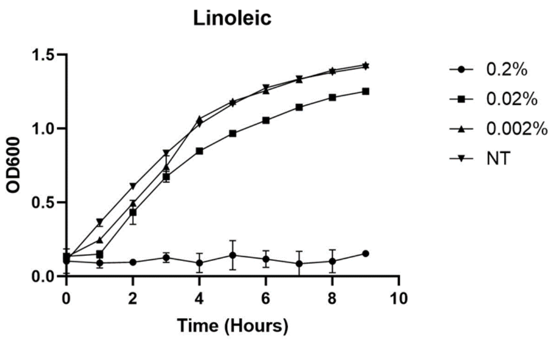
Linoleic
OD600
Time (Hours)
0.2%
0.02%
0.002%
NT

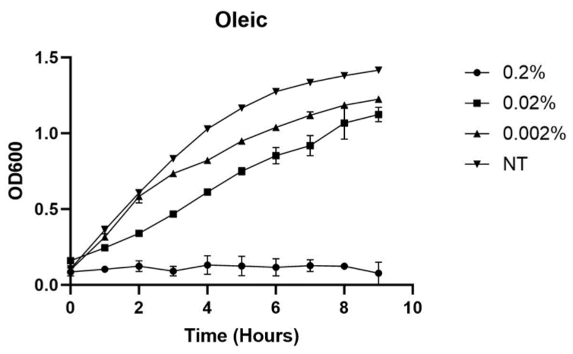
Oleic
OD600
Time (Hours)
0.2%
0.02%
0.002%
NT

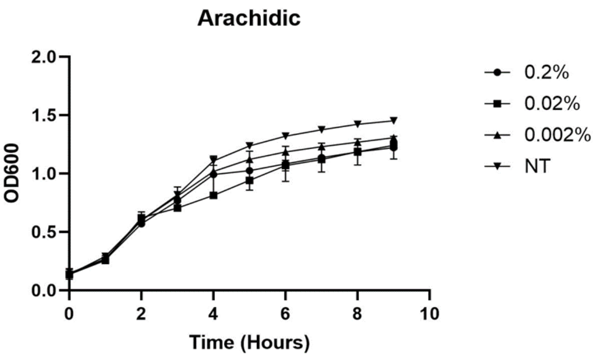
Arachidic
OD600
Time (Hours)
0.2%
0.02%
0.002%
NT

Fig. S3

***icsA* transcription after treatment with the most effective VirF inhibitory FAs. Linoleic acid (LI) was used as negative control (* p < 0.01, **p <0.05)**

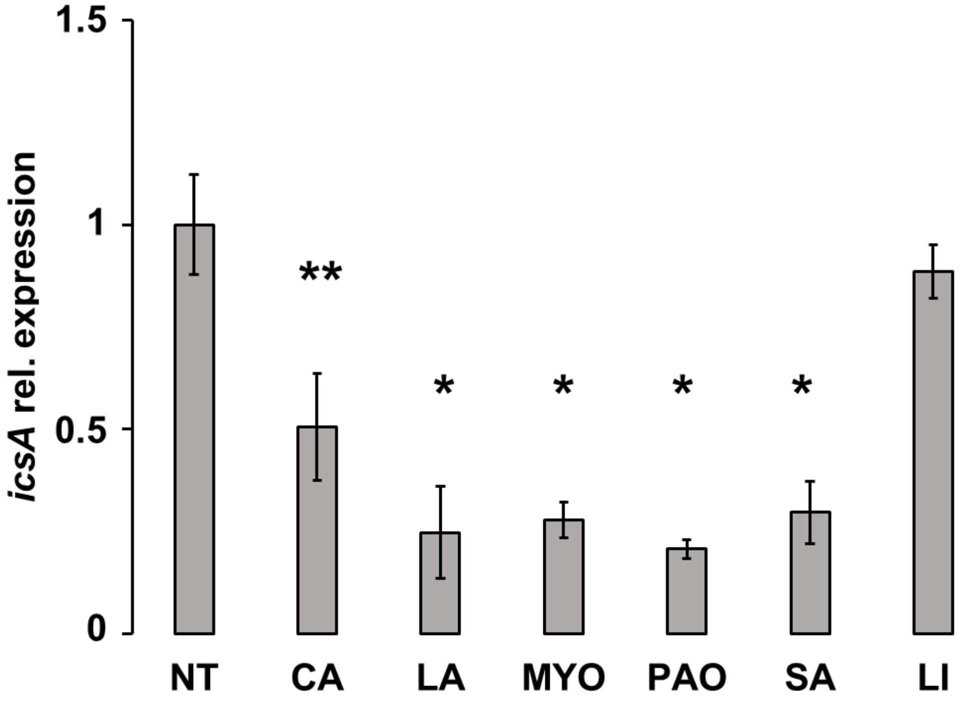

Fig. S4

Western blot showing the wild-type and mutated VirF proteins in untreated samples.

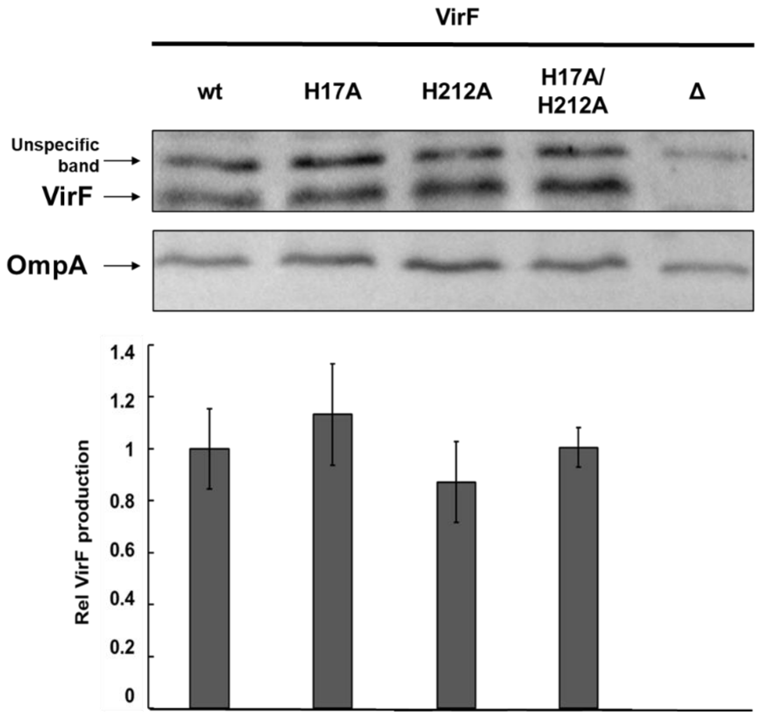

Fig. S5

Congo Red plate assay

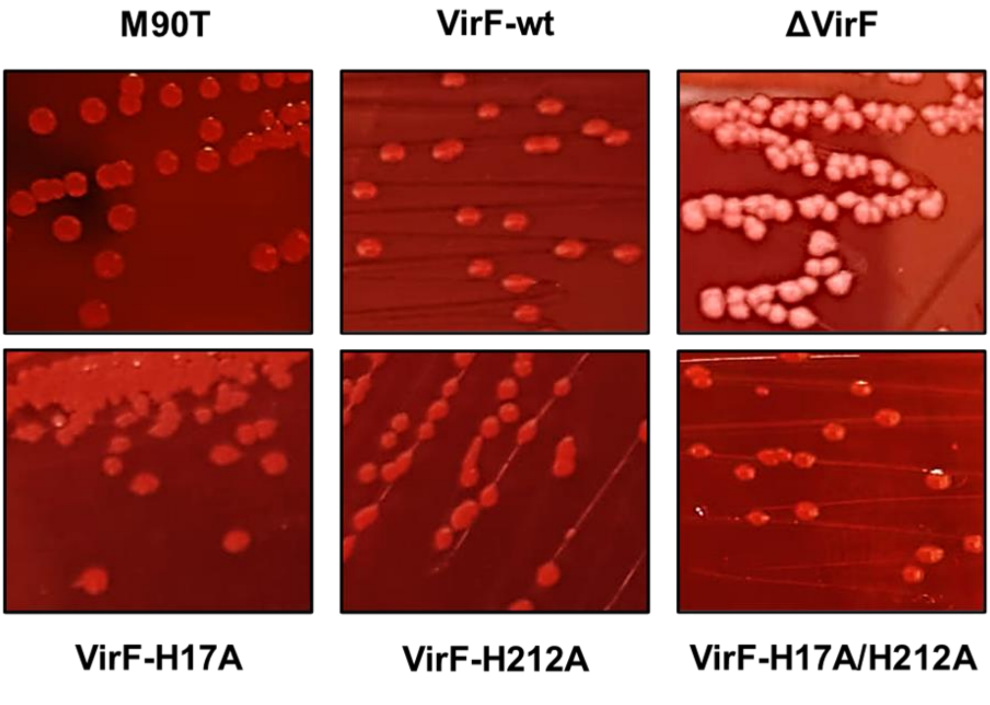

Fig. S6

ΔC_t values of M90T untreated samples (C_t*virB*-C_t*nusA*) expressing the w.t. and mutated VitF protein.

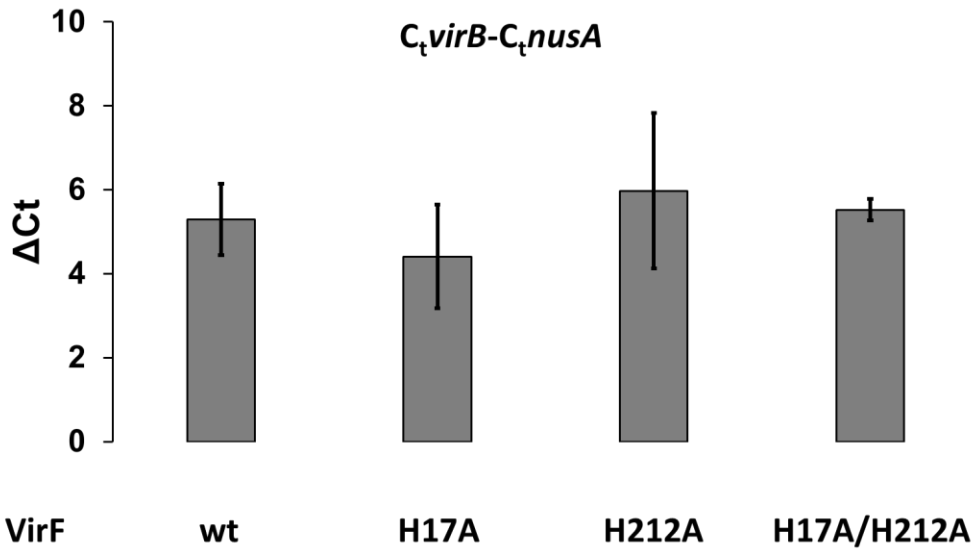

Fig. S7

DPIPA analysis: assessment of DNA saturation point.

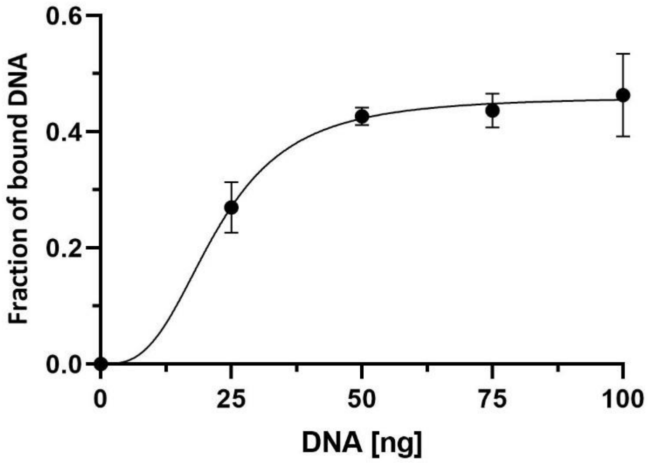

	CT
EC_{50}, µM<br>(95% CI)	22,21<br>(16,75 to 27,67)

REFERENCES SUPPLEMENTAL MATERIAL

1. Sansonnetti PJ, Kopecko DJ, Formal SB. 1982. Involvement of a plasmid in the invasive ability of Shigella flexneri. Infect Immun 35:852.
2. Di Martino ML, Romilly C, Wagner EGH, Colonna B, Prosseda G. 2016. One Gene and Two Proteins: a Leaderless mRNA Supports the Translation of a Shorter Form of the Shigella VirF Regulator. mBio 7:e01860-16.
3. Leuzzi A, Di Martino ML, Campilongo R, Falconi M, Barbagallo M, Marcocci L, Pietrangeli P, Casalino M, Grossi M, Micheli G, Colonna B, Prosseda G. 2015. Multifactor Regulation of the MdtJI Polyamine Transporter in Shigella. PLoS One 10:e0136744.
4. Datsenko KA, Wanner BL. 2000. One-step inactivation of chromosomal genes in Escherichia coli K-12 using PCR products. Proc Natl Acad Sci U S A 97:6640–6645.
5. Uzzau S, Figueroa-Bossi N, Rubino S, Bossi L. 2001. Epitope tagging of chromosomal genes in Salmonella. Proc Natl Acad Sci U S A 98:15264.
6. Adler B, Sasakawa C, Tobe T, Makino S, Komatsu K, Yoshikawa M. 1989. A dual transcriptional activation system for the 230 kb plasmid genes coding for virulence-associated antigens of Shigella flexneri. Mol Microbiol 3:627–635.
7. Prosseda G, Falconi M, Giangrossi M, Gualerzi CO, Micheli G, Colonna B. 2004. The virF promoter in Shigella: more than just a curved DNA stretch. Mol Microbiol 51:523–537.
8. Shen MY, Sali A. 2006. Statistical potential for assessment and prediction of protein structures. Protein Sci. 15:2507–2524.
